# Different renal manifestations associated with very early onset pediatric inflammatory bowel disease: case report and review of literature

**DOI:** 10.1186/s12882-021-02358-2

**Published:** 2021-04-22

**Authors:** A. Angeletti, S. Arrigo, A. Madeo, M. Molteni, E. Vietti, L. Arcuri, M. C. Coccia, P. Gandullia, G. M. Ghiggeri

**Affiliations:** 1 Division of Nephrology, Dialysis, and Transplantation, IRCCS Istituto Giannina Gaslini, Genoa, Italy; 2 Pediatric Gastroenterology Unit, IRCCS Istituto Giannina Gaslini, Genoa, Italy; 3 Department of Pathology, IRCCS Istituto Giannina Gaslini, Genoa, Italy; 4 Laboratory of Molecular Nephrology, IRCCS Istituto Giannina Gaslini, Genoa, Italy

**Keywords:** Inflammatory bowel disease, Extraintestinal manifestations, IgA nephropathy, Granulomatous interstitial nephritis, Acute kidney injury, Case report

## Abstract

**Background:**

Inflammatory bowel diseases are characterized by chronic inflammation of the gastrointestinal tract. In particular, Crohn disease and ulcerative colitis represent the two most common types of clinical manifestations. Extraintestinal manifestations of inflammatory bowel diseases represent a common complications, probably reflecting the systemic inflammation. Renal involvement is reported in 4–23% of cases. However, available data are limited to few case series and retrospective analysis, therefore the real impact of renal involvement is not well defined.

**Case presentation:**

We report the case of a 10-years old male affected by very early onset unclassified-Inflammatory bowel diseases since he was 1-year old, presenting with a flare of inflammatory bowel diseases associated with acute kidney injury due to granulomatous interstitial nephritis. Of interest, at 7-year-old, he was treated for IgA nephropathy.

To our knowledge, no previous reports have described a relapse of renal manifestation in inflammatory bowel diseases, characterized by two different clinical and histological phenotypes.

**Conclusions:**

The link between the onset of kidney injuries with flares of intestinal inflammation suggest that nephritis maybe considered an extra-intestinal manifestation correlated with active inflammatory bowel disease. However, if granulomatous interstitial nephritis represents a cell-mediated hypersensitivity reaction than a true extraintestinal manifestation of inflammatory bowel diseases is still not clarified. We suggest as these renal manifestations here described may be interpreted as extraintestinal disorder and also considered as systemic signal of under treatment of the intestinal disease.

## Background

Crohn disease (CD) and ulcerative colitis (UC) represent the two most common inflammatory bowel diseases (IBD), however in paediatric subjects 15–20% of IBD are defined unclassified (IBD-U), due to an overlapping features of CD and UC [1]. 4–20% of paediatric IBD have an early presentation (< 6 year old) and part of them may be correlated with a primary immunodeficiency [2].

Despite the high widespread of the disease worldwide, the definitive pathological mechanisms are still far to be completely clarified. Growing studies reported as an impairment of T cell function may represent a pivotal pathway both in CD and in UC [3, 4].

Extraintestinal manifestations of IBD, probably reflecting the systemic inflammation, are common and occur in approximately one third of patients [5]. Renal involvement is described in around 4–23% of patients with IBD [6], but reports are limited to few case series and retrospective analysis. Proteinuria, hematuria, and/or impairment of renal function may reflect urological disorders (i.e. urinary calculi, fistulas and obstruction) or parenchymal kidney disease [6]. IgA nephropathy is considered the typical histological finding on renal biopsy of patients with IBD, but also tubulointerstitial nephritis have been reported. Tubulointerstitial nephritis is often caused by drugs (5-aminosalicylates) [7], but it may represent a true extraintestinal manifestation of IBD itself and occur in the absence of drugs administration.

Herein, we describe the case of a 10-years old male affected by very early onset (VEO) unclassified-IBD (IBD-U) since he was 1-year old, already treated for IgA nephropathy at the age of 7, and now presenting with a flare of IBD associated with acute kidney injury due to granulomatous interstitial nephritis.

To our knowledge, no previous reports have described this dual renal pathology in IBD.

## Case presentation

We describe a 10-year old boy who was diagnosed with pancolitis VEO IBD-U at the age of 1 and treated with oral mesalazine from the beginning of the disease. The patient received a total of three gut biopsy that confirmed IBD-U.

Over the years, he presented a steroid sensitive disease course, refractory to azathioprine (May 2010- Feb 2012) and methotrexate (Feb 2012- Jun 2012). Afterwards, he obtained clinical and mucosal remission on thalidomide (June 2012- Sep 2017), that was stopped due to progressive peripheral neuropathy, considered as adverse event of thalidomide [8]. Therefore it was shifted to the chimeric anti-TNF IgG1 monoclonal antibody infliximab (June 2018- Sep 2020), resulting in a progressive resistance to treatment despite the increased dose (10 mg/kg/month). Oral mesalazine was administered from Jan 2010 to Jan 2016 and then stopped after the first episode of hematuria with persisting microhematuria. Topically mesalazine was administered for limited time in 2010 and in 2020. Immunological screening (antinuclear antibodies, anti-neutrophil cytoplasmic antibodies, anti-Saccharomyces cerevisiae antibodies, anti-mitochondrial antibodies, liver-kidney microsomal antibodies, rheumatoid factor) was normal and genetic panel for immunodefinciencies related to VEO-IBD resulted negative.

**Fig. 1:**
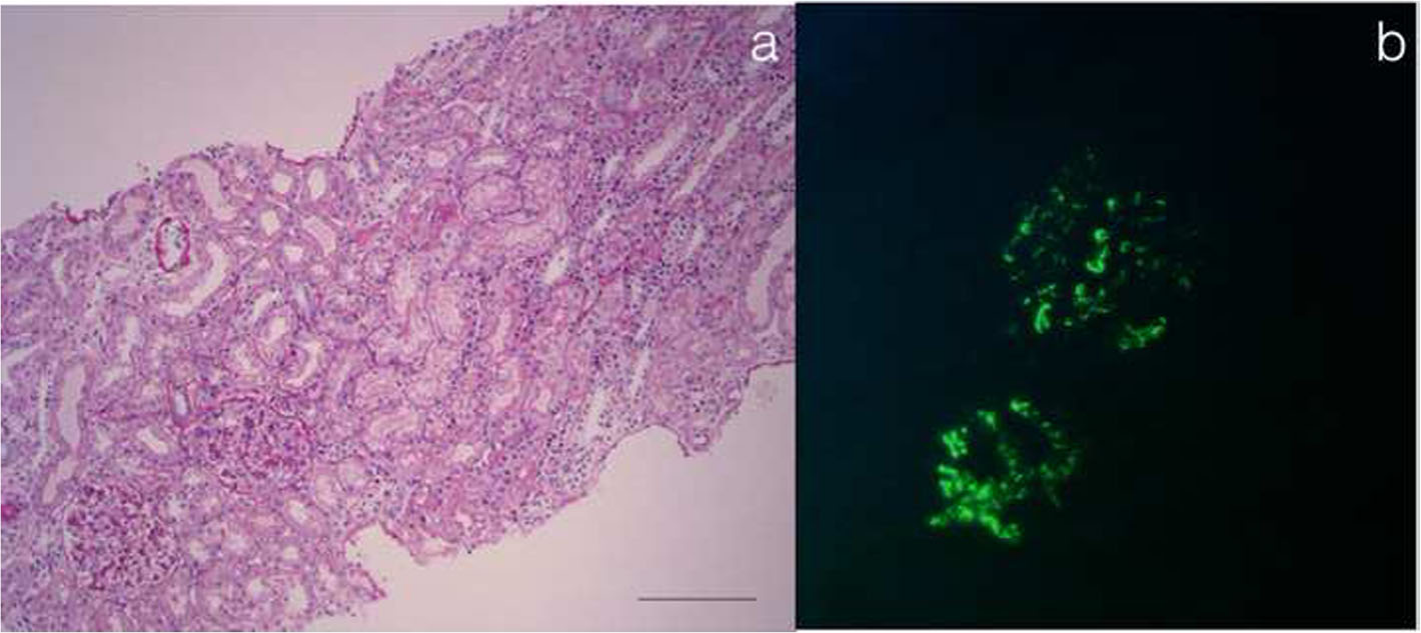
IgA Glomerulonephritis. **a** At light microscopic mesangial hypercellularity (M1 > 50% of glomeruli). No endocapillary hypercellularity (E0). No segmental glomerulosclerosis (S0) and no tubular atrophy/interstitial fibrosis (T0). At light microscopy there were no interstitial nephritis. (hematoxylin and eosin, original magnification × 200) **b** Immunofluorescence positive for IgA in mesangium (3+). Immunofluorescence for C3, C4d, IgM and IgG resulted negative (not shown)

In February 2016, a first episode of hematuria and dysuria was reported: urine examination tested positive for red blood cells and proteins not in nephrotic range, while urine colture and renal ultrasound were negative. Renal function, erythrocyte sedimentation rate, reactive-c-protein and serum complement components C3 and C4 were normal. In September 2017, the persistence of microhematuria and mild proteinuria (< 1 g/die) led to a kidney biopsy that revealed IgA nephropathy with moderate mesangial hypercellularity (M1,E0,S0,T0 according to the Oxford Classification [9, 10]) (Fig. 1). Steroidal bolus, according to the randomized clinical trial proposed by Pozzi et al. [11] were administrated from November 2017 to May 2018 with complete remission of renal disease. Urine sample were monitored every 6 months resulting always negative until January 2020, when macrohaematuria was once again reported during an episode of fever and diarrhea. Renal function was normal with no proteinuria.

After high steroid therapy, IBD was well controlled with Infliximab and rectal mesalazine plus steroid for 2 months. From May 2020 recurrent episodes of isolated fever every 2 to 3 days were reported. The temperature rise was well controlled with paracetamol and never associated with abdominal pain or diarrhea. Macrohaematuria appeared again by August 2020.

**Fig. 2:**
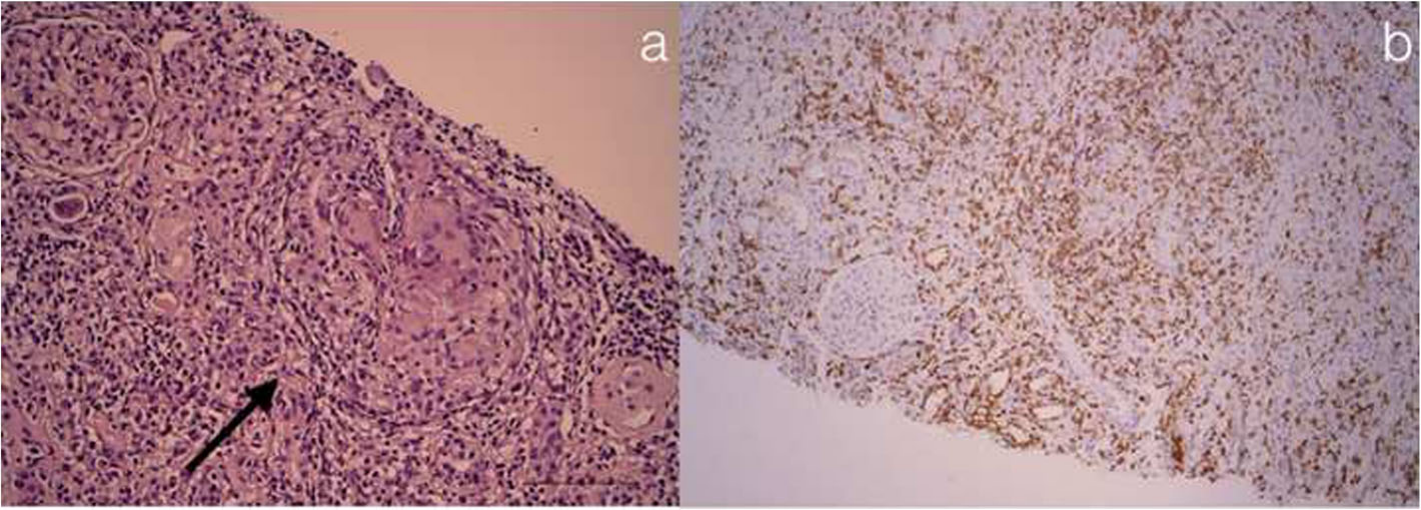
Non-Necrotizing Granulomatous Interstitial Nephritis. **a** Granulomatous interstitial nephritis with interstitial infiltration by mononuclear cells and noncaseating granulomas with multinucleated giant cells (arrows) (hematoxylin and eosin and PAS, both original magnification × 200), with negative Ziehl-Neelsen and Grocott stains (not shown). We performed immunohistochemistry for CD45, CD3 (**b**), CD4, CD8, CD20, CD68, CD138 and CD31. The interstitium is affected by a mixed cellular inflammatory infiltrate. CD3 T lymphocytes are the most represented elements, with CD4 and CD8 positive subpopulation, B lymphocytes in minority part, various plasma cells, histiocytes, plurinuclear giant cells and some granulocytes neutrophils

In September 2020, the boy was admitted to Gastroenterology department of our Paediatric Hospital with a relapse of IBD-U. Blood exams revealed acute kidney injury with increased levels of creatinine (1,5 mg/dL), urea (44 mg/dL) and limited proteinuria 0,3 g/24 h. Reactive-C-protein was 2.9 mg/dl, serum eosinophils and IgE in norma range. At urine microscopy we detected 20 red blood cells per field and less than 20% were dysmorphic; leucocytes (20–25/filed) were also present. Renal Ultrasound revealed an hyper-echogenity of renal parenchima, suggesting interstitial chronic lesions. The patient underwent a second renal biopsy, described in Fig. 2**,** that revealed a non-necrotizing granulomatous interstitial nephritis, characterized by a prevalence of T lymphocytes. Tuberculosis and infective causes were excluded. Moreover, patients resulted positive for for IgG against *Bartonella henselae*. Therefore we performed a rtPCR for *Bartonella henselae* in renal tissue that resulted negative.

As therapy, we administered oral steroid at the dose of 60 mg/m^2^ for 1 month, than tapered to 40 mg/m^2^ for further 4 weeks. As long-acting treatment, considering the relapse of IBD and the granulomatous interstitial nephritis occurred during therapy with infliximab, we started adalimumab (160 mg at baseline, 80 mg at week 2 following with 40 mg/week). At 6 months of follow up, serum creatinine was 0.7 mg/dl (eGFR 81 ml/min) and no clinical manifestations of IBD are reported.

## Review of the literature

Table 1 summarizes the main literature reporting renal histology in patients affected by IBD. Overall, in accordance with the first report by Hubert et al. [16] in 1984, following studies confirmed IgA nephropathy as the most common type of histological diagnosis. Few patients had granulomatous tubulo-interstitial nephritis that, at the time, were correlated with the exposure to aminosalicylate, but a clear pathological mechanism was not proposed. To the best of our knowledge, a relapse of renal manifestation, characterized by dual renal pathology, has never been previously reported.

**Table 1:** Main clinical studies reporting renal histology in Inflammatory Bowel Disease

References	N	Renal Histology	Comments
Ambruzs et al. [12]	45 CD 38 UC	24% IgA Nephropathy; 19%Interstitial Nephritis; 12% Nephrosclerosis; 8% Acute Tubular Injury; 7% Proliferative GN; 4% Minimal change disease;	• Prevalence of IgAN significantly higher in patients with IBD than in healthy population • Association with HLA-DR1described in both IBD and IgAN • All patients with interstitial nephritis were previously exposed to aminosalicylates
Jang et al. [13]	7 CD	5 IgA Nephropathy; 1 Henoch-Schönlein purpura; 1 No alterations;	
Archimandritis et al. [14]	1 CD	Interstitial nephritis with granulomas	• Full recovery after proctocolectomy
Izzedine et al. [15]	4 CD	4 Interstitial Nephritis	• All diagnosed before administration of mesalazina • Progression to end-stage renal failure in 3 patients • Granulomas were identified in 2 patients
Hubert et al. [16]	1 CD 1 UC	IgA Nephropathy	• Restoration of renal findingsafter treatment for IBD
Ridder et al. [17]	1 UC	Membranous Nephropathy	• Presentation with intestinal manifestation • Improvement after therapy
Pohjonen et al. [18]	14 CD 14 UC 7 UND	7 IgA Nephropathy; 2 Membranous Nephropathy; 2 IgM GN; 4 acute interstitial nephritis; 4 chronic interstitial nephritis;	• All patients with interstitial nephropathy had an history of mesalazina administration
Ota et al. [19]	1 CD	acute tubulointerstitial nephritis	• After the discontinuation of IFX, renal abnormalities resolved

*CD* Chron disease, *GN* glomerulonephrities, *IBD* inflammatory bowel disease, *UC* ulcerative colitis, *UND* undetermined

## Discussion and conclusion

We describe the case of a 10-year male affected by VEO IBD-U since he was 1-year old and already treated for IgA nephropathy when he was 7-year old. After several years and a good clinical course, he presented with acute kidney injury due to granulomatous interstitial nephritis.

IgA nephropathy is the most common renal manifestation in patients affected by IBD. Other types of glomerulonephritis in IBD, including membrano-proliferative glomerulonephritis, minimal change disease, membranous nephropathy, anti-glomerular basement membrane glomerulonephritis and C3 glomerulopathy have been only rarely reported and cannot be considered as potential associations [12, 13, 17].

Giving the relatively high incidence of subclinical IgA nephropathy in the general population, some authors hypothesized that IBD would be coincidental and act only exacerbating primary IgA nephropathy [20]. However, the strict correlation between the onset of glomerulonephritis with flare of intestinal inflammation and the improvement of renal disease achieved with control of IBD inflammation, suggest that glomerulonephritis represents more an extra-intestinal manifestation than a casual association. Different mechanisms are probably implicated, such as cytokine-induced inflammation leading to both glomerular and intestinal tract inflammation and damage [21]. Increasing evidence support as the mucosal immune system is involved in primitive IgA nephropathy. The mesangial deposits in IgA nephropathy are mainly polymeric IgA1, for which the mucosal immune system represents the major source. Infections in the respiratory or gastrointestinal tract with a triggered mucosal immune response are commonly associated with IgA nephropathy. Therefore, intestinal mucosal break and loss of surface integrity, during bowel inflammation, may lead to an increased exposure of antigens with consequent increase in circulating IgA immunoglobulins, that can subsequently be deposited in the glomeruli [6].

Moreover, a genetic association with HLA-DR1 in both IBD and IgA nephropathy [22–25] and the correlation between histological resolution of IgA nephropathy [26, 27] after IBD treatment [7, 28] or proctocolectomy [14], strengthen the clinical relation. In fact, treatment of IBD inflammation, with concomitant IgA nephropathy, usually results in improvement and often normalization of renal function that is an indirect sign of histological remission of glomerulonephritis. Renal manifestations were successfully treated with cycles of intravenous steroid in accordance with the classical approach for the primary IgA nephropathy, but this might have been secondary to successful treatment of IBD relapse [29]. The second kidney biopsy (Fig. 2), performed during a further colitis relapse 3 years later, documented chronic mesangial lesions, with a degeneration of some glomeruli that were already compromised, with no deposition of IgA at immunofluorescence.

As extraintestinal manifestations of IBD tubular injuries have a reported prevalence between 2 and 16% among renal manifestations [15, 30, 31]. In some cases, tubulointerstitial nephritis is diagnosed simultaneously with IBD prior the beginning of any therapy, and often recovers after an effective IBD treatment [32]. In this sense, it could be considered a real extraintestinal disorder correlated with active IBD (fever, abdominal pain and/or diarrhea) [33]. Granulomatous interstitial nephritis, characterized by an inflammatory infiltrate containing at least one epithelioid histiocytes mixed with lymphocytes with or without multinucleated giant cells, are reported in limited experiences and often associated to recent or past exposure to aminosalicylates [12, 18]. However, if granulomatous interstitial nephritis represents a cell-mediated hypersensitivity reaction than a true extraintestinal manifestation of IBD is not clarified. In our report, oral mesalazine was administered several years before the kidney failure and in the previous renal biopsy there were no signs of interstitial nephritis (Fig. 1). Moreover, the chimeric anti-TNF IgG1 monoclonal antibody infliximab was associated to renal complications such as glomerulonephritis [34, 35] or, in limited cases, to the development of acute kidney injury due to tubule-interstitial nephritis [19, 36]. Infliximab-induced tubule-interstitial nephritis may develop after long-term administration [19] and the recovery of kidney function was reported 2–3 months after the withdrawal of infliximab, in accordance with the half-life of the drug [36].

It is fundamental distinguish drug-induced tubule-interstitial nephritis from extraintestinal lesions due to IBD, that is usually accompanied by gastro-intestinal symptoms. In the present case, we report the detection of high level of serum creatinine corresponded with an acute phase of IBD. Therefore, the detection of a granulomatous interstitial nephritis was suggestive of a true extraintestinal manifestation.

In conclusion, we described the case of a 10-years old male affected by VEO IBD-U, previously treated for IgA nephropathy at the age of 7, and now presenting with a relapse of IBD-U associated with acute kidney injury due to granulomatous interstitial nephritis. We suggest that these renal manifestations may be interpreted as extraintestinal disorder and also considered as systemic signal of under treatment of the intestinal disease.

## Data Availability

The datasets used and/or analysed during the current study available from the corresponding author on reasonable request.

## Abbreviations

CD: Crohn disease
IBD: Inflammatory bowel diseases
IBD-U: Unclassified-IBD
UC: Ulcerative colitis
VEO: Very early onset
